# UV Light-Assisted Synthesis of Highly Efficient Pd-Based Catalyst over NiO for Hydrogenation of *o*-Chloronitrobenzene

**DOI:** 10.3390/nano8040240

**Published:** 2018-04-14

**Authors:** Weidong Jiang, Bin Xu, Guangyin Fan, Kaiming Zhang, Zhen Xiang, Xiaoqiang Liu

**Affiliations:** 1 School of Chemistry and Environmental Engineering, Sichuan University of Science & Engineering, Zigong 643000, China; xbjwd@suse.edu.cn (B.X.); kmzhang@suse.edu.cn (K.Z.); zxiang@suse.edu.cn (Z.X.); xqliu@suse.edu.cn (X.L.); 2 Key Laboratory of Green Catalysis of Sichuan Institute of High Education, Sichuan University of Science & Engineering, Zigong 643000, China; 3 College of Chemistry and Materials Science, Sichuan Normal University, Chengdu, 610068, China; fanguangyin@cwnu.edu.cn

**Keywords:** UV-light irradiation, Pd/NiO catalyst, o-chloronitrobenzene, hydrogenation

## Abstract

Supported Pd-based catalyst over active nickel oxide (NiO) was repared using the impregnation method companying with UV-light irradiation. Moreover, the catalytic performance of the obtained Pd-based catalysts was evaluated towards the hydrogenation of *o*-chloronitrobenzene (*o*-CNB). Observations indicate that the as-prepared UV-irradiated Pd/NiO catalyst with a mole fraction 0.2% (0.2%Pd/NiO) has higher activity and selectivity in the *o*-CNB hydrogenation. Especially, UV-light irradiation played a positive role in the improvement of catalytic activity of 0.2%Pd/NiO catalyst, exhibiting an excess 11-fold activity superiority in contrast with non-UV-irradiated 0.2%Pd/NiO catalyst. In addition, it was investigated that effects of varied factors (i.e., reaction time, temperature, *o*-CNB/Pd ratio, Pd loading, hydrogen pressure) on the selective hydrogenation of *ο*-CNB catalyzed by UV-irradiated 0.2%Pd/NiO catalyst. Under the reaction conditions of 60 °C, 0.5 h, 1 MPa H_2_ pressure, 100% conversion of *o*-CNB, and 81.1% *o*-CAN selectivity were obtained, even at high molar ratio (8000:1) of *o*-CNB to Pd.

## 1. Introduction

Aromatic amines, especially aromatic haloamines, are typical of important raw materials for pharmaceuticals, pigments, dyes, and agrochemicals [1,2,3]. Catalytic hydrogenation of aromatic nitro compounds shall be the most common approach for the production of aromatic amines [4,5,6,7]. In the last two decades, large amounts of researches involve the homogeneous [8,9] and heterogeneous hydrogenation reduction [10,11,12,13] of nitro compounds. When comparing with homogeneous reaction, heterogeneous hydrogenation displays dominating character in most of its industrial processes, and thereby it has attracted more attention in designing special catalysts for the heterogeneous hydrogenation reactions [14]. In the designs of heterogeneous catalysts, varied supports with dazzling morphologies were selected as candidates, such as carbon-based materials [15,16,17,18], metallic or non-metallic oxidates [19,20,21], and polymer [22,23].

As we know, zero-valent metal on supported catalysts acted as real catalytic species in the catalytic hydrogenation reactions [24]. Therefore, immobilized metal catalysts generally underwent a reduction process of cationic metals using different reduction methods, such as chemical reduction [25] and hydrogen gas reduction [26]. However, the two kinds of reduction methods possess their inherent defects, representing potential explosive for hydrogen gas as well as environmental hazard and operated dangerous for chemical reductants. Therefore, scientists are devoted to finding other reduction strategies that are green and sustainable, such as the reduction in EtOH employing it as solvent and reducing agent under a nitrogen atmosphere in the autoclave, or under microwave employing ethylene glycol or alcohols as solvent and reducing agent [27,28,29,30]. Herein, we especially highlight Enrico Borgarello’s work [31], providing an interested and convenient alternative (UV light-irradiated reduction) for the above-mentioned strategies in the reduction of metal cations. Along this line of consideration, in our previous report [32], UV-light-irradiated reduction was testified to take effect for the preparation of a Pd-based catalyst over α-Fe_2_O_3_, which exhibited good activity in the catalytic hydrogenation of *o*-chloronitrobenzene (*o*-CNB).

**Scheme 1:**
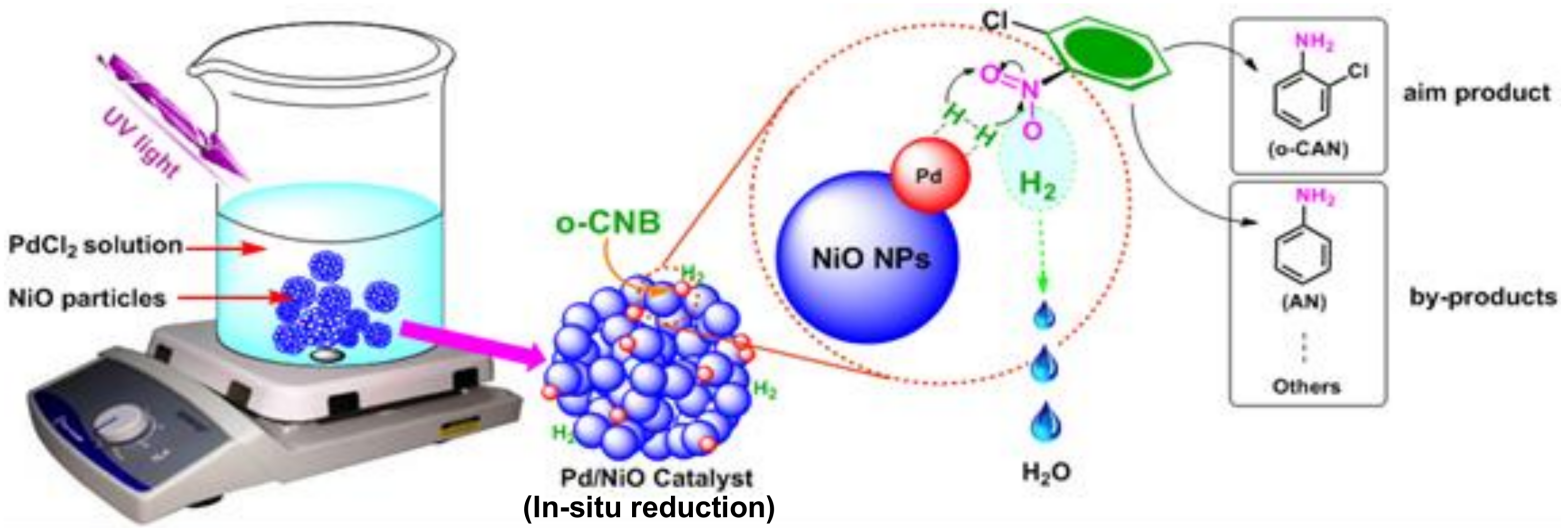
Sketch map of the preparation of UV-light-irradiated Pd-based catalyst over NiO and the evaluation of its reactivity toward the o-chloronitrobenzene (*o*-CNB) hydrogenation.

To further judge the reliability and validity of this reported method, in this paper, we attempt to prepare another Pd-based catalyst (UV-irradiated Pd/NiO) over fresh active nickel oxide (NiO) particles using UV light-irradiation reduction, and systematically evaluated its reactivity in the selectively hydrogenation of *o*-CNB. Scheme 1 illustrates the preparation of UV-light irradiated Pd/NiO catalyst and the sketch of *o*-CNB hydrogenation promoted by the as-prepared Pd catalyst. Contrast studies concerning both of Pd/NiO catalysts confirmed our anticipation that UV-light irradiation indeed performed a particular function in the improvement of catalytic activity of Pd/NiO catalyst. Moreover, the screened UV-irradiated Pd/NiO catalyst still maintained high activity at great substrate/catalyst (Pd content) mole ratios ranged from 4000 to 9000. 

## 2. Materials and Methods

### 2.1. Reagents and Chemicals

All of the reagents and chemicals are of analytical grade and were used as received without further purification. PdCl_2_·2H_2_O as Pd source was purchased from Chengdu Kelong Chemical Ltd. (Chengdu, China). Authoritative samples of *o*-CNB, *o*-chloroaniline (*o*-CAN), and aniline (AN) are products of Sigma-Aldrich Co. (Shanghai, China) Active NiO powders were freshly prepared in our lab according to the method, described as follows. 

### 2.2. Preparation of Active NiO Powders

Aqueous solution of nickel(II) nitrate hexahydrate (0.1 mol/L) was prepared in deionized water. Using BT-200B-type constant-current pump (Shanghai Huixi Analysis Instrument Co., Ltd., Shanghai, China), ammonia solution as precipitant was added dropwise into the Ni(NO_3_)_2_·6H_2_O solution with speed of 0.5 drops per second. pH value of the mixed solution was kept at 8 during the addition of ammonia solution. After finishing the adding of ammonia solution, the residual ammonia was removed from the mixed solution. The resulting solution was allowed to stand in air, and then was filtrated under vacuum companying with a washing operation with anhydrous ethanol for at least three times. Afterwards, the obtained powders were dried under low pressure at 80 °C for four hours. Furthermore, the dried solid underwent a grinding operation and was calcinated in a muffle furnace at 500 °C for three hours, thus producing the desired active NiO powders.

### 2.3. Preparation of Pd-Based Catalysts over NiO Particles

The as-prepared active NiO powders were added to an aqueous solution of PdCl_2_·2H_2_O (0.2% theoretic mole fraction of loaded Pd). The resulting mixture was stirred thoroughly under ultraviolet irradiation [31] (30 W) with a wavelength of 365 nm for 24 h. Afterwards, the mixture was filtered under low pressure, meanwhile washed with deionized water. The powdered solids were dried in vacuum at 80 °C for 4 h. Ultimately, the desired 0.2% Pd/NiO catalyst was collected by grinding the dried solids.

### 2.4. Catalyst Characterizations

Scanning electron microscope (SEM) with an energy-dispersive X-ray spectroscopy detector (EDS) (Bruker AXE, Karlsruhe, Germany) was recorded on a VEGA 3SBU instrument (Tescan Co., Brno, Czech Republic). Low resolution transmission electron microscope (TEM) images were determined on a JEOL JEM 1200EX (Akishima-shi, Tokyo, Japan) working at 100 kV. High-resolution TEM images (HRTEM) and selected-area electronic diffraction (SAED) patterns were recorded with FEI Tecnai G2 F20 S-Twin (Hillsboro, OR, USA) working at 200 kV. The crystalline phases of the as-prepared Pd-based catalysts and the reused catalyst were recorded on a Bruker D2-PHASER X-ray diffractometer (XRD) (Bruker AXE, Karlsruhe, Germany) with nickel-filtered Cu Kα radiation (λ = 1.5417 Å, 40 kV, 25 mA) in the 2θ zone of 10°–90°. The Pd content of the Pd/NiO catalyst was collected on Spectro Arcos ICP-OES (Germany Spectro Analysis Co., Ltd., Kleve, North Rhine-Westphalia, Germany). X-ray photoelectron spectroscopy (XPS) was recorded on Thermo ESCALAB 250XI spectrometer (ThermoFisher Scientific Co., Ltd., Waltham, MA, USA). BET surface areas were determined on a SSA-4200 Specific Surface Area and Porosity Analyzer (Beijing Builder Electronic Technology Co., Ltd., Beijing, China) with 30% *v*/*v* N_2_/H_2_ flow using pure N_2_ (99.99%) as an internal standard. 

### 2.5. Catalyst Evaluation

The resulted 0.2% Pd/NiO catalyst was evaluated for the selective hydrogenation of *o*-CNB in 100 mL capacity of GYF-type stainless-steel autoclave (Xingtianyu Experimental Instrument Co., Ltd., Chengdu, China) with PTFE lining, thermocouple, and a mechanical stirrer. Reaction reagents, including *o*-CNB (5 mmol), Pd/NiO catalyst (20 mg) and 5 mL of absolute ethanol as solvent were introduced into the reactor before each run. Inner air was displaced with high purity compressed hydrogen (99.999%) for at least three times. The hydrogenation reaction of *o*-CNB started after the reaction mixture was heated to the pre-set temperature. The *o*-CNB hydrogenation was lasting for 30 min. After filtering the hydrogenation residues, the organic filtrate was used to analyze the hydrogenation products. 

### 2.6. Product Analysis 

The filtrate above-mentioned was analyzed using a SC-3000B-204 gas chromatography (Chuanyi Analyzer Co., Ltd., Chongqing, China) equipped with a hydrogen flame ionization detector (HFID) and a SE-54 capillary column (size: 30 m × Ф 0.53 mm). The column temperature was adjusted to 150 °C. Meanwhile, the vaporization chamber temperature and detector temperature were respectively controlled at 200 and 250 °C. Quantitative analysis of the resulting hydrogenation products was performed by the area normalization method with a HW-2000 chromatography workstation (Qianpu Software, Nanjing, China). 

## 3. Results

### 3.1. Preparation and Characterizations of Various Pd-Based Catalysts

**Figure 1:**
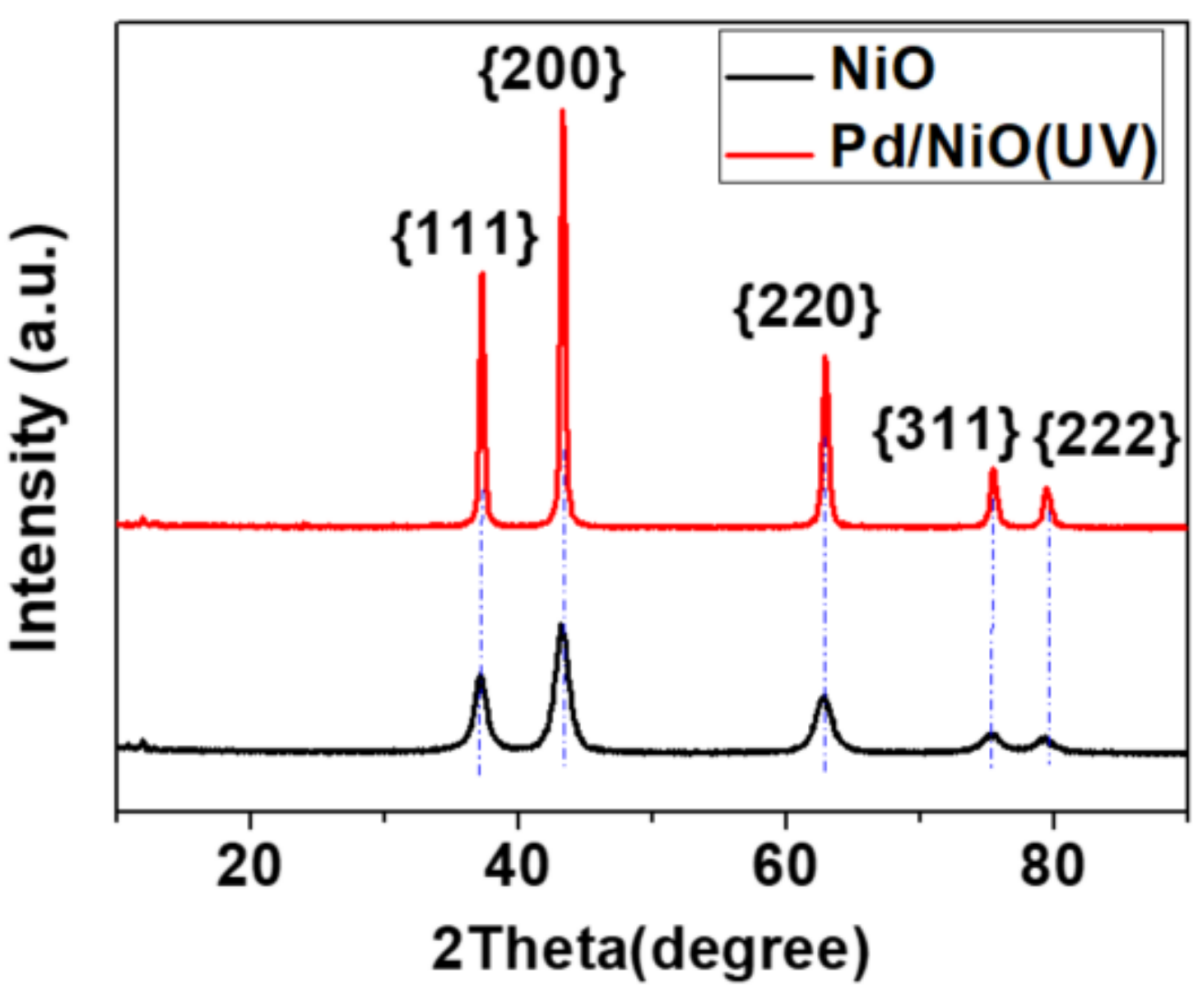
X-ray diffraction patterns of NiO and UV-irradiated Pd/NiO catalyst.

Catalytic hydrogenation requires the use of a metal particle in a finely divided state, where both the size and the shape are crucial parameters that must be mediated to maximize their functions [33]. Hence, structural characterizations of the obtained Pd/NiO catalyst were performed here by XRD, SEM, TEM, and XPS. Figure 1 indicates the XRD patterns of NiO carrier and Pd/NiO composites, from which we can probe two kinds of vital information. Firstly, those characteristic peaks located at 37.15°, 43.20°, 62.85°, 75.36°, and 79.25° (2θ) match well with the scattering signals from (111), (200), (220), (311), and (222) planes (JCPDS card no. 47-1049) of NiO crystals. Moreover, it was observed that XRD patterns of UV-irradiated Pd/NiO catalyst display stronger and sharper characterized peaks in contrast with those of the nickel oxide carriers. This implies that UV-irradiated Pd/NiO samples possess relatively well crystalline relative to the NiO carrier. Another aspect shows that Pd crystal phase was not detected with XRD, probably resulting from no metallic Pd(0) species or highly small particle size as well as the high dispersion of zero-valent Pd species on the surface of NiO particles.

**Figure 2:**
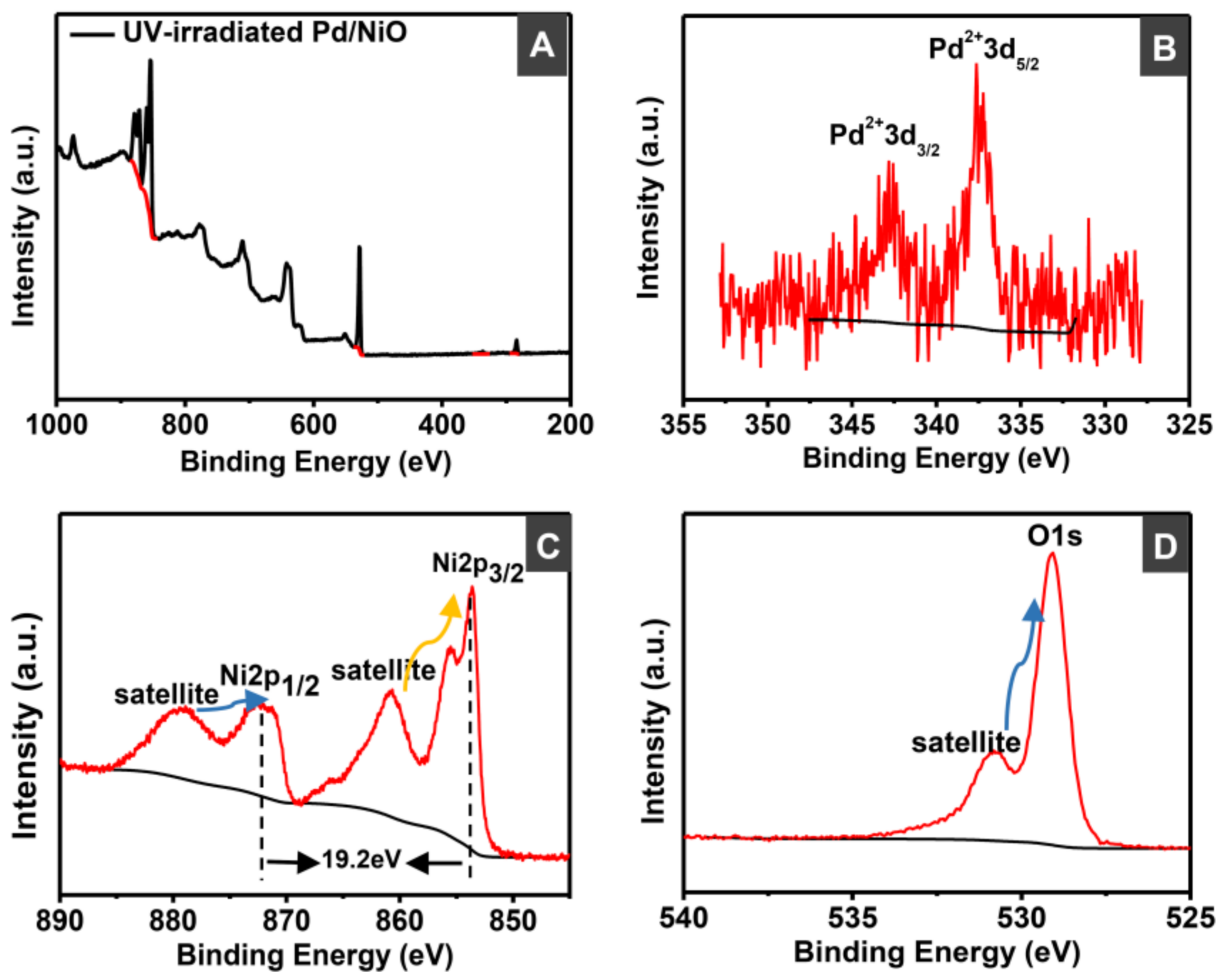
X-ray photoelectron spectroscopy (XPS) spectra of UV-irradiated Pd/NiO catalyst.

Chemical valences of exterior elements and the surface chemical composition of the UV-irradiated Pd/NiO catalyst were determined by the XPS technique (Figure 2. Figure 2A gives a wide XPS spectra of this Pd-based catalyst. As shown in Figure 2B, binding energies (BE) of Pd3d_5/2_ and Pd3d_3/2_ in UV-irradiated Pd/NiO catalyst, which, respectively, locates at about 337.0 and 343 eV, are approximately 2.0 eV higher than those distinctive values of zero-valent Pd, as reported in the literature [34]. The BE values demonstrate the existence of bivalent Pd cations, which is not agreement with our anticipation. Ultimately, the XPS information provides further evidence for Pd2+ species inside UV-irradiated Pd/NiO catalyst. Synchronously, it also testifies that the real cause for no XRD characterized peak (see Figure 1) of zero-valent Pd is its inexistence not the other two possibilities (e.g., high dispersion and very small particle size). Moreover, the almost same binding energy (See Support Information, Figure S1) of Pd species indicates that Pd species is Pd^2+^ cations in the non-UV-irradiated catalyst. XPS spectra (Figure 2C) of the Ni 2p region give discernible features, suggesting the existence of NiO form. Namely, the Ni 2p spectrum is split into two spin-orbit doublets as main peaks that are located at ~854 eV for Ni 2p_3/2_ and ~873 eV for Ni 2p_1/2_, respectively. Corresponding satellite peaks of above-mentioned main peaks are observed at ~861 eV and ~880 eV, which is in line well with previous reports [35]. Relative value between their main peak and satellite peak is almost the same one (approximately 19 eV) as each other. Binding energy of the O 1s at ~529.1 eV is assigned to the lattice oxygen of the nickel oxide sample [35]. 

**Figure 3:**
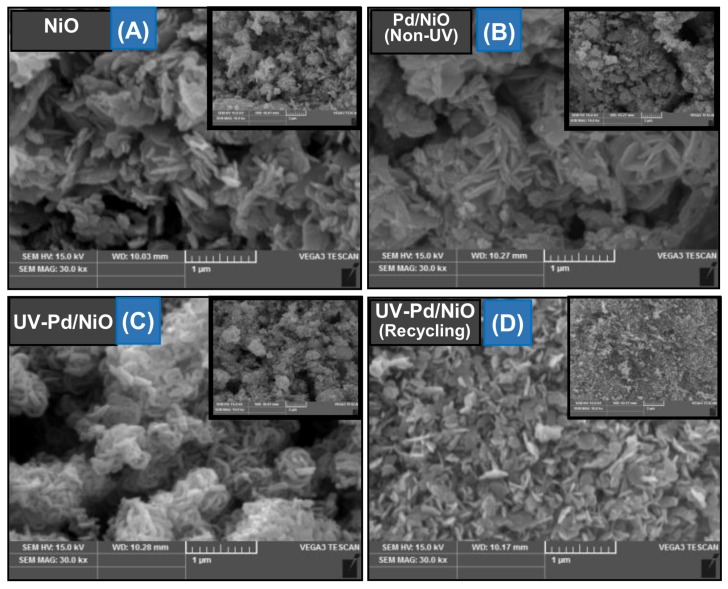
Scan electron microscope images of varied samples including NiO carrier (**A**) and Pd-based catalysts (UV-irradiation, **B**; non-UV irradiation, **C**; reused samples, **D**) at an enlarged scale of 30,000 times. Meanwhile, corresponding SEM images of the same samples were inserted at a smaller scale of 10,000 times.

SEM characterizations respectively shown in Figure 3 provide us with precise surface morphology of NiO and Pd/NiO catalysts. SEM images that are given here show that the support NiO powders are stacked aggregates of NiO nanosheets, and this kind of stacking is irregular (Figure 3A). Interestingly, some flower-like spheroidal aggregates of Pd/NiO without UV-irradiation (Figure 3B) appeared, in which many visible double-layer petal textures transferred from the originated single-layer sheet. This change reveals that the introduced Pd is able to motivate the reaggregation of NiO. Further, magic transformation for the morphology of UV-irradiated Pd/NiO catalyst was recorded (Figure 3C). Each near-spherical Pd/NiO aggregate was stacked with relatively thicker and fringe-smooth NiO sheets. Moreover, cave-like morphology gradually appears on the surface of NiO sheets. Under UV irradiation, double-layer petal textures of non-UV-irradiated Pd/NiO dramatically transferred to a kind of interest mutual ring-linkage structure. This interested structural change probably results from the cooperative induction of UV-light irradiation and Pd even if the correlative influencing mechanism is not clear now. Remarkably, collected catalyst sample after three runs exhibited a morphology change producing divided slices without obvious flower-like aggregation (Figure 3D). Thereupon, an interesting change in surface morphology testifies to the positive role of UV-light irradiation in the preparation of NiO-loaded Pd catalyst even if UV irradiation did not work in the Pd reduction. This UV-induced morphology change probably contributes to the much higher activity of UV-irradiated Pd-based catalyst.

**Figure 4:**
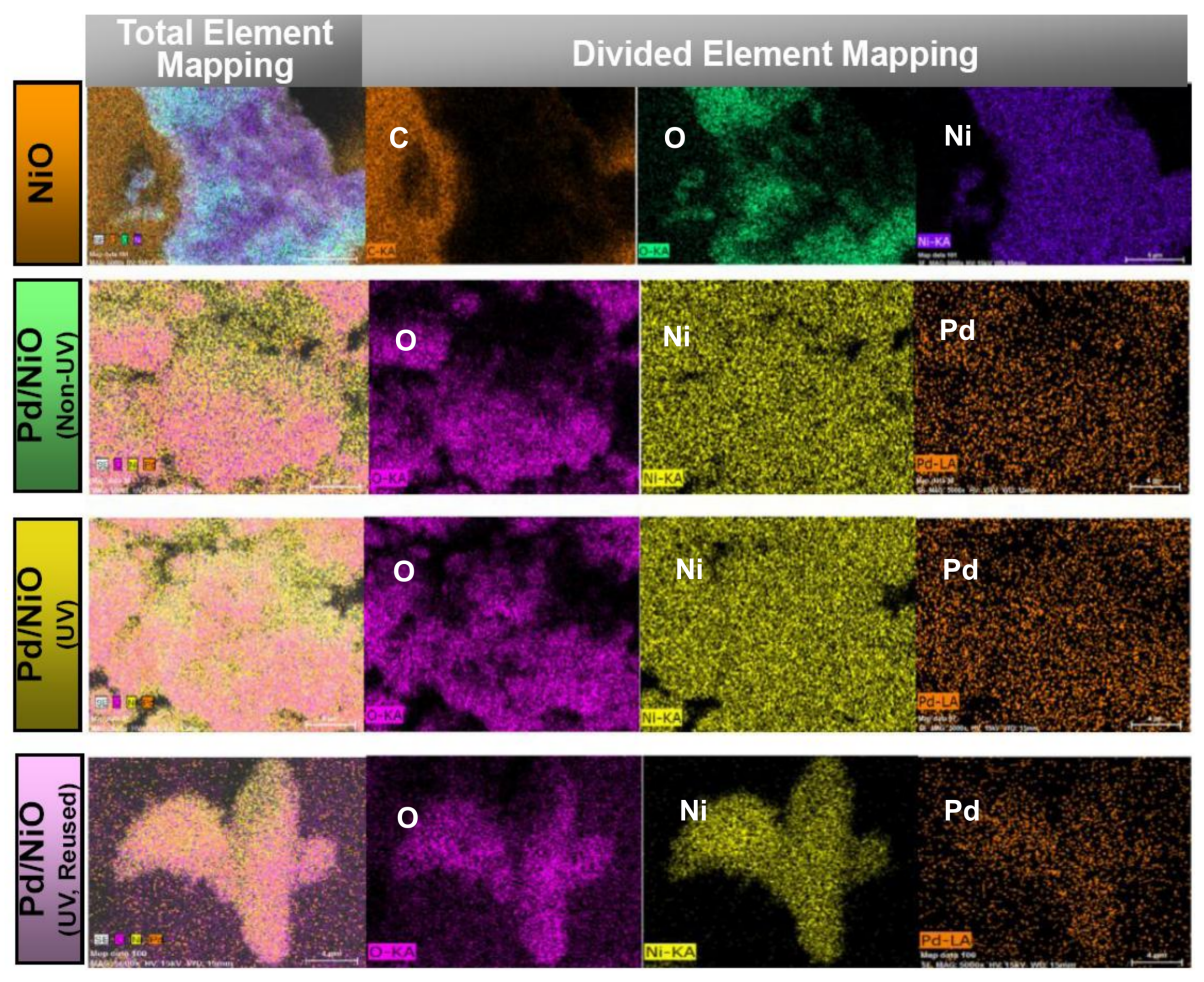
Element mapping of all samples including NiO carrier and Pd-based catalysts. Carbon element mapping indicates carbon conduction adhesive.

**Figure 5:**
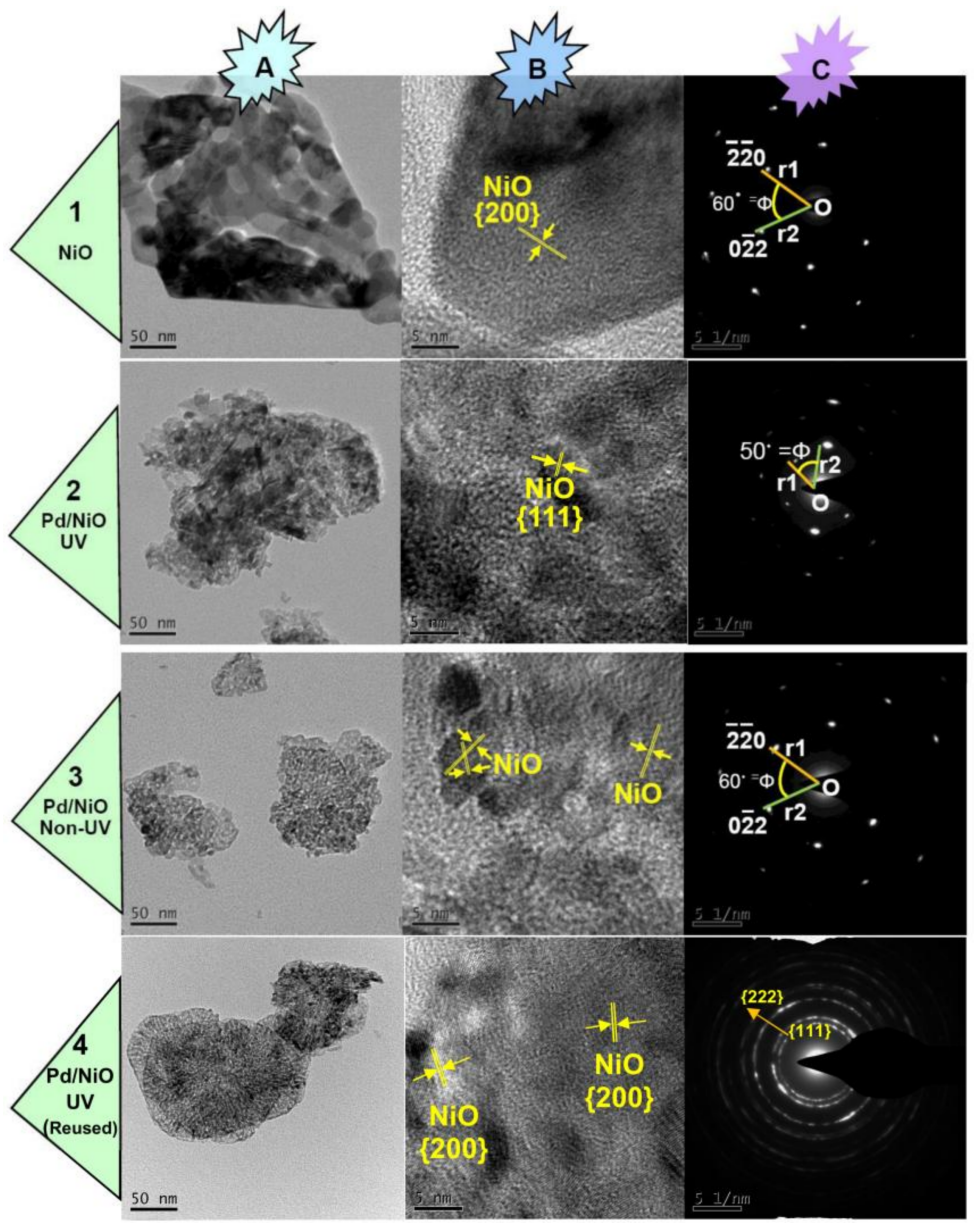
Transmission electron microscope (TEM, HRTEM) images (A*n*, B*n*) and selected-area electronic diffraction (SAED) patterns (C*n*) of varied samples including NiO and Pd-based catalysts (UV or non-UV irradiation, reused one after the fourth run).

Roughly, EDS examination (not shown here) shows that the Pd loading (about 0.19%) is highly in agreement with the theoretic value (0.2%), demonstrating no obvious loss of Pd during the preparation process of Pd/NiO catalyst. Element mapping (Figure 4) also shows that Pd species indeed successfully absorbed on the support NiO. Figure 5 depicts TEM (column A: A1–A4) and HRTEM images (column B: B1–B4) of three samples. As shown in column B (i.e., B1, B2, B3, B4) of Figure 5, the interplanar spacing of approximate 0.24 nm and 0.20 nm is in accordance with the lattice spacings of the {111} and {200} planes of NiO [36], thus confirming the NiO phase. As for all of the catalyst samples, unfortunately, there is no characteristic value of lattice spaces corresponding to the zero-valent Pd. As shown in column C of Figure 5, furthermore, the selected-area electronic diffraction (SAED) patterns of the above-mentioned samples indicate a better crystallization degree of three initial samples. Howbeit, polycrystalline phases were observed from the SAED image (Figure 5C4) of the reused catalyst (four tests), suggesting an aggregation behavior of UV-irradiated Pd/NiO catalyst. Diffraction rings from inside to outside are assigned to {111}, {200}, {220}, {311}, and {222} lattice planes of *fcc* NiO, respectively. The SAED image in Figure 5C3 of non-UV-irradiated Pd/NiO is almost the same as that of pure *fcc* NiO sample shown in Figure 5C1, revealing that no active Pd atom appears in this Pd/NiO sample without UV-irradiation. As shown in Figure S2, particle-size distribution represents all of samples are not uniform, especially for non-UV-irradiated sample. Also, Figure S2 reveals that, to some extent, UV-irradiation is in favor of relatively narrow size distribution around ~50 nm. But, non-UV-irradiated sample displays a very broad range of particle sizes. Figure 5C2 also indicates no Pd crystallic phase in the UV-irradiated Pd/NiO catalyst, implying that UV-light irradiation unsuccessfully played its unique function in the reduction of Pd^2+^ ion. In fact, the acquired species information from SAED patterns is in conformity with that obtained information from the HRTEM images of these samples. However, it is an indubitable fact that UV-irradiated Pd/NiO exhibited higher hydrogenation activity of *o*-CNB in this work. Herein, we believe that highly efficient hydrogenation of *o*-CNB by the UV-irradiated Pd/NiO sample probably results from the formation of active atomic Pd by an in-situ hydrogen-induced reduction of Pd^2+^ with hydrogen gas that comes from an external filling and an ethanol reforming route [37]. Importantly, the unique change of catalyst’s morphology originated from UV-irradiation probably plays a crucial and synergistic role in the obvious improvement of hydrogenation reactivity of Pd/NiO catalyst in contrast with non-UV-irradiated sample.

### 3.2. Positive Effect of UV-Light Irradiation

**Figure 6:**
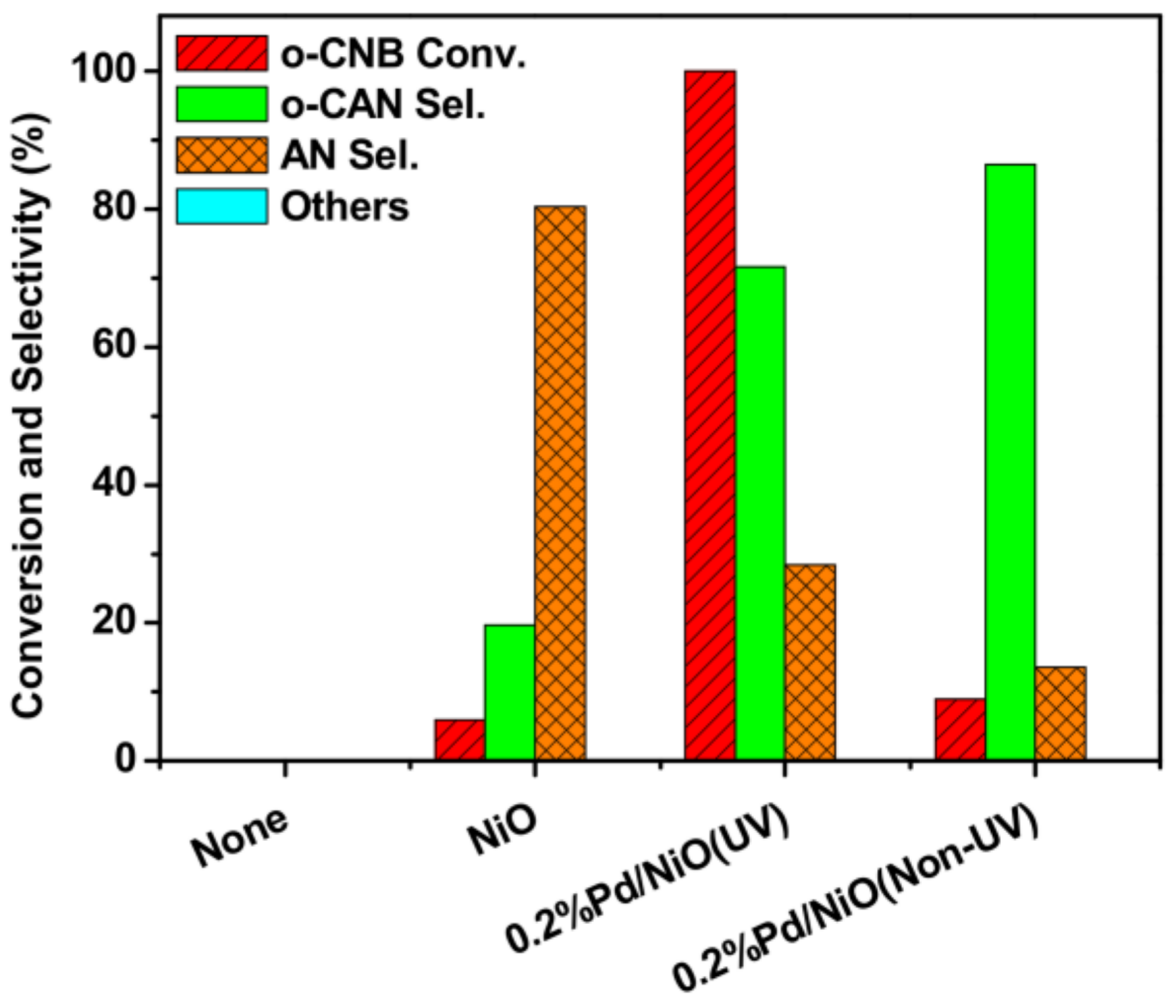
Catalytic hydrogenation of *o*-CNB in different catalytic systems. Conditions: 60 °C, 0.5 h, *P*(H_2_) = 1 MPa, *n_o_*_-CNB_/*n*_Pd_ = 5000:1, *V*_ethanol_ = 5 mL.

In previous reports, the UV-irradiation method was applied in the activation of some heterogeneous catalysts [32]. Taking into account this strategy, we studied the hydrogenation performance of *o*-CNB over an UV-irradiated Pd/NiO catalyst for comparing its activity with that non-UV-irradiated Pd/NiO catalyst. Figure 6 shows the different hydrogenation activity of *o*-CNB in the presence of various catalyst samples. Clearly, UV-irradiated Pd/NiO catalyst displayed much better catalytic activity in comparison with that of the Pd/NiO catalyst without UV-irradiation operation, indicating an excess of 11-fold superiority in activity even if *o-*CAN selectivity slightly decreased. On the whole, catalytic capacity of Pd/NiO is obviously improved under UV-light irradiation during the preparation process of the desired Pd/NiO catalyst. Namely, under the same operated conditions, *o*-CAN yield (71.6%), which results from the 0.2%Pd/NiO(UV)-induced *o-*CNB hydrogenation is 61.7-fold and 11-fold higher than those of NiO- and 0.2% Pd/NiO(non-UV)-catalyzed reaction, respectively.

Distinguishable activities of UV- or non-UV-irradiated 0.2% Pd/NiO catalysts may be provided available information from above-mentioned structural characterizations. The XPS spectra in Figure 2 fail to provide evidence for the formation of zero-valent Pd in all of the catalyst samples, including UV-irradiated and non-UV-irradiated Pd/NiO catalysts. Noteworthy, good hydrogenation reactivity of *o*-CNB by UV-irradiated Pd/NiO sample probably results from the in-situ formation of atomic Pd by a hydrogen-induced reduction of Pd^2+^ cations in catalytic system, as illustrated in the fourth paragraph of Section 3.1. In addition, the changed surface morphology of UV-irradiated Pd/NiO catalyst plays a positive role in the increase in its activity when compared to the non-UV-irradiated one. BET analysis data (Table 1) denote that 0.2% Pd/NiO(UV) particles possess a smaller surface area of 25.3 m^2^·g^−1^ with the pore size of about 14 nm. In contrast, non-UV-irradiated 0.2%Pd/NiO has a significant surface area of 104.7 m^2^·g^−1^ with a smaller pore size of 3.6 nm. Overall, all of the catalyst samples present small surface area and pore volume, which can be supported by their low dispersion morphology (see Figure 3). When comparing with the larger surface area of non-UV-irradiated Pd/NiO, the smaller surface area of UV-irradiated Pd/NiO particles may be originated from the relatively compact structure shown in Figure 3C. In general, larger specific surface area facilitates the chemisorption and physical absorption of reagents on the surface of supported catalysts inspiring much faster reaction rates [38]. In fact, catalytic activity of the non-UV-irradiated Pd/NiO was neglected in comparison with the UV-irradiated one. These evidences clearly demonstrate that, in the current work, the higher activity of UV-irradiated Pd-based catalyst mainly originated from the appearance of in-situ reduced Pd(0) and the possible synergistic function of UV-induced morphology change, even if the potential reason for the positive function is not clear temporarily. In the case of the non-UV-irradiated sample, on the contrary, it is probably the secondary factor for improving activity that the better adsorptive ability toward the reaction reagents due to larger surface area of Pd/NiO sample without UV irradiation. Consequently, we think that UV-light irradiation possesses a vital function in the preparation of heterogeneous catalysts with good catalytic reactivity. Thereupon, UV-irradiated 0.2%Pd/NiO catalyst was selected as the desired catalyst for subsequent hydrogenation experiments in this work. 

### 3.3. Effect of Pd Loading

**Figure 7:**
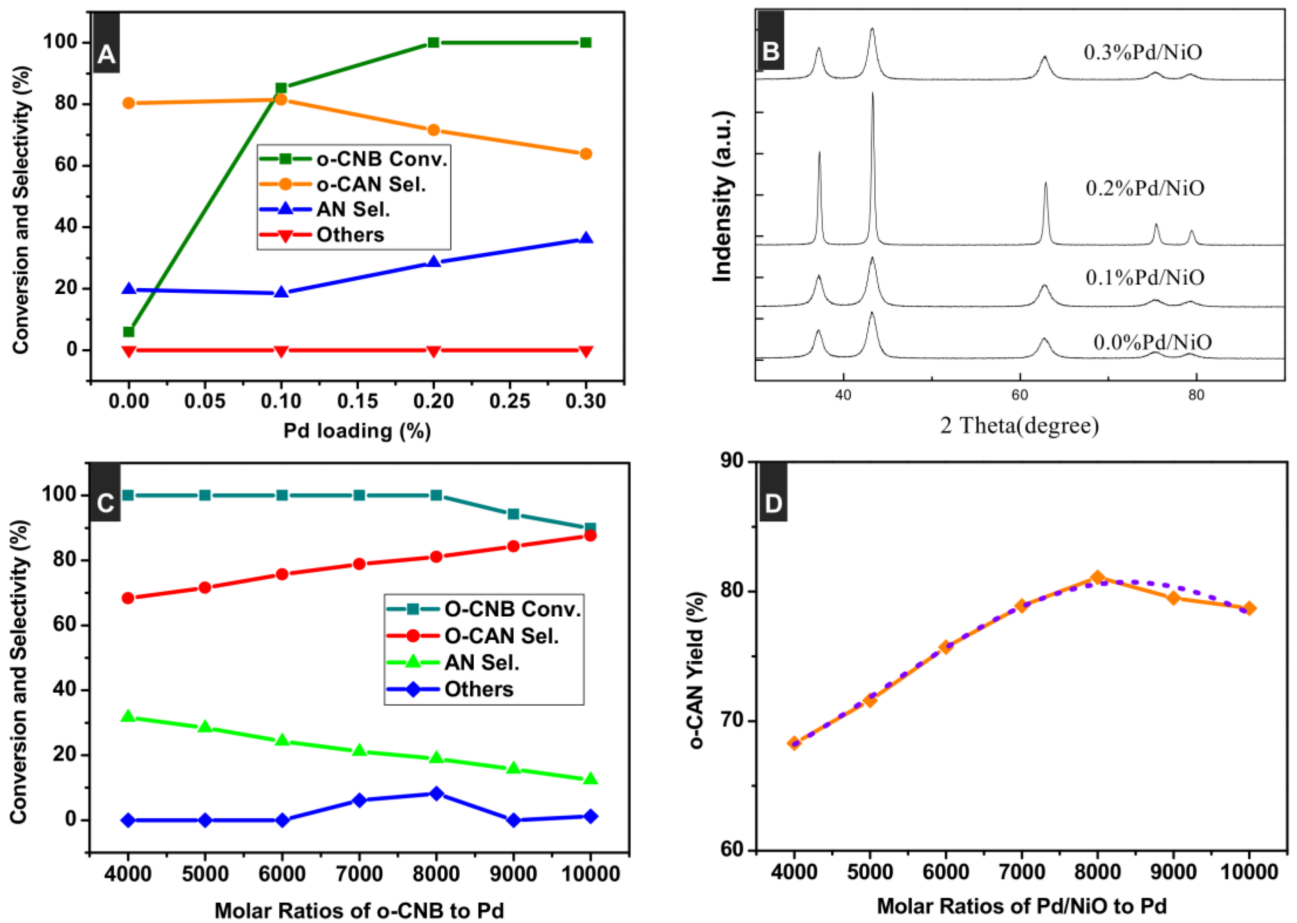
Effects of Pd content (**A**) and *o*-CNB /Pd molar ratios (**C**,**D**) upon the *o*-CNB hydrogenation. XRD patterns of NiO-loaded Pd catalysts with varied Pd content is shown in (**B**). In (**D**), orange solid line shows the actual trend of *o*-CAN yield meanwhile dash line is its Gaussian fitting curve. Conditions: (**A**): 60 °C, 0.5 h, *P*(H_2_) = 1 MPa, *m*_cat._ = 20 mg, *n_o_*_-CNB_/n_Pd_ = 5000:1, *V*_ethanol_ = 5 mL. (**C**,**D**): 60 °C, 0.5 h, *P*(H_2_) = 1 MPa, *V*_ethanol_ = 5 mL.

As for heterogeneous hydrogenation over supported catalysts with active noble metals, the content of the loaded metal is also an important influencing factor for the hydrogenation reactivity of the supported metal catalysts [39]. As a consequence, we decided to study the dependence of the Pd/NiO catalyst’ reactivity upon the Pd loadings under selected conditions (Figure 7A). The hydrogenation of *o*-CNB only obtained a 5.9% conversion of *o*-CNB and 80.3% *o*-CAN selectivity over NiO without the addition of Pd. Along with the increasing Pd content, furthermore, the *o*-CNB conversion sharply increased at 0.1% of Pd level. When the Pd loading content was up to 0.2% or 0.3%, *o*-CNB completely converted to hydrogenated products with a 100% conversion. The highest yield of the target product (*o*-CAN) was obtained when UV-irradiated 0.2% Pd/NiO (UV) served as a catalyst. Unexpectedly, on the whole, the selectivity of *o*-CAN showed a downward trend companying with an increased AN selectivity. This fact probably comes from the high hydrogenolysis activity of Pd for C-Cl bond, leading to the concomitant dechlorination of *o*-CNB, which produces more AN [5].

As seen from Table 1, in the case of adopting UV-light irradiation, there is no obvious difference in the surface textural properties between three Pd/NiO catalysts with varying Pd content. Their surface textural parameters are almost identical to those that support NiO. In the absence of UV-irradiation, the specific surface area of Pd/NiO sample was highly enlarged, and its pore volume became somewhat bigger than other catalysts. These unusual phenomena possibly imply that UV-light irradiation, rather than the Pd addition, plays a vital role in the controlling of surface morphology of the as-prepared Pd-based catalysts. XRD patterns shown in Figure 7B indicate that 0.2% Pd/NiO catalyst possesses a better crystallization degree in contrast to the others. Possibly, there are favorable exposed crystallographic facets [40] for the adsorption/activation of the reaction molecules and the products desorption in the *o*-CNB hydrogenation to *o*-CAN over 0.2% Pd/NiO catalyst, which may be one of the major causes for the preponderant activity of the 0.2% Pd/NiO catalyst. By the way, the influencing mechanism of UV-light irradiation on morphology will be especially documented in our further work. Herein, we selected a moderate content (0.2%) of Pd component for the subsequent hydrogenation experiment of *o*-CNB.

### 3.4. Effect of Molar Ratios of o-CNB to Pd

In most of previous reports, a relatively lower level of substrate concentration was adopted in heterogeneous hydrogenation reactions [41]. If keeping at higher substrate concentration, the lower conversion of substrate can generally be observed. In the present work, we attempted to investigate the hydrogenation process of *o*-CNB at a high concentration level of *o*-CNB relative to Pd content. Figure 7C shows the variations of *o*-CNB conversion and *o*-CAN selectivity as a function of the molar ratios of *o*-CNB to Pd. It was clearly found that 0.2% Pd/NiO constantly kept an efficient activity in the operated range of *o*-CNB/Pd molar ratios from 4000 to 8000, exhibiting 100% *o*-CNB conversion. Higher yield (~79.5%) of *o*-CAN could still achieved even at a much great *o*-CNB /Pd molar ratio (9000). Another aspect showed a desired increase of *o*-CAN as the aimed product with the increase in the *o*-CNB/Pd ratio. Fortunately, the observed results are comparable to the commercial Pd/C [42,43].

However, the *o*-CNB hydrogenation displayed a declined tendency along with the further increase in *o*-CNB concentration as the *o*-CNB/Pd molar ratio exceeds 8000. The highest total yield of *o*-CAN reached at 8000 of molar ratio, depicting a bell-shaped curve (Figure 7D). This behaviour reflects the spatial inhibition [44] of the absorption of hydrogen molecules on the active Pd sites due to the competitive absorption of more substrate molecules. To some extent *o*-CNB molecules with high concentration serve as diluent for efficient reductant (H_2_), which led to a decreasing H_2_ concentration on catalytic sites. Thus, the real concentration of active hydrogen atoms on the surface of Pd atoms was reduced, depressing the hydrogenation reaction of *o*-CNB. Those previous works [45,46] also provided a highly similar tendency for the varying of hydrogenation rate with initial substrate concentration. In view of this influencing factor, the hydrogenation reactions by heterogeneous catalysts shall require fitting the initial concentration of substrates so that it obtains much better hydrogenation efficiency. 

### 3.5. Effect of Reaction Time

**Figure 8:**
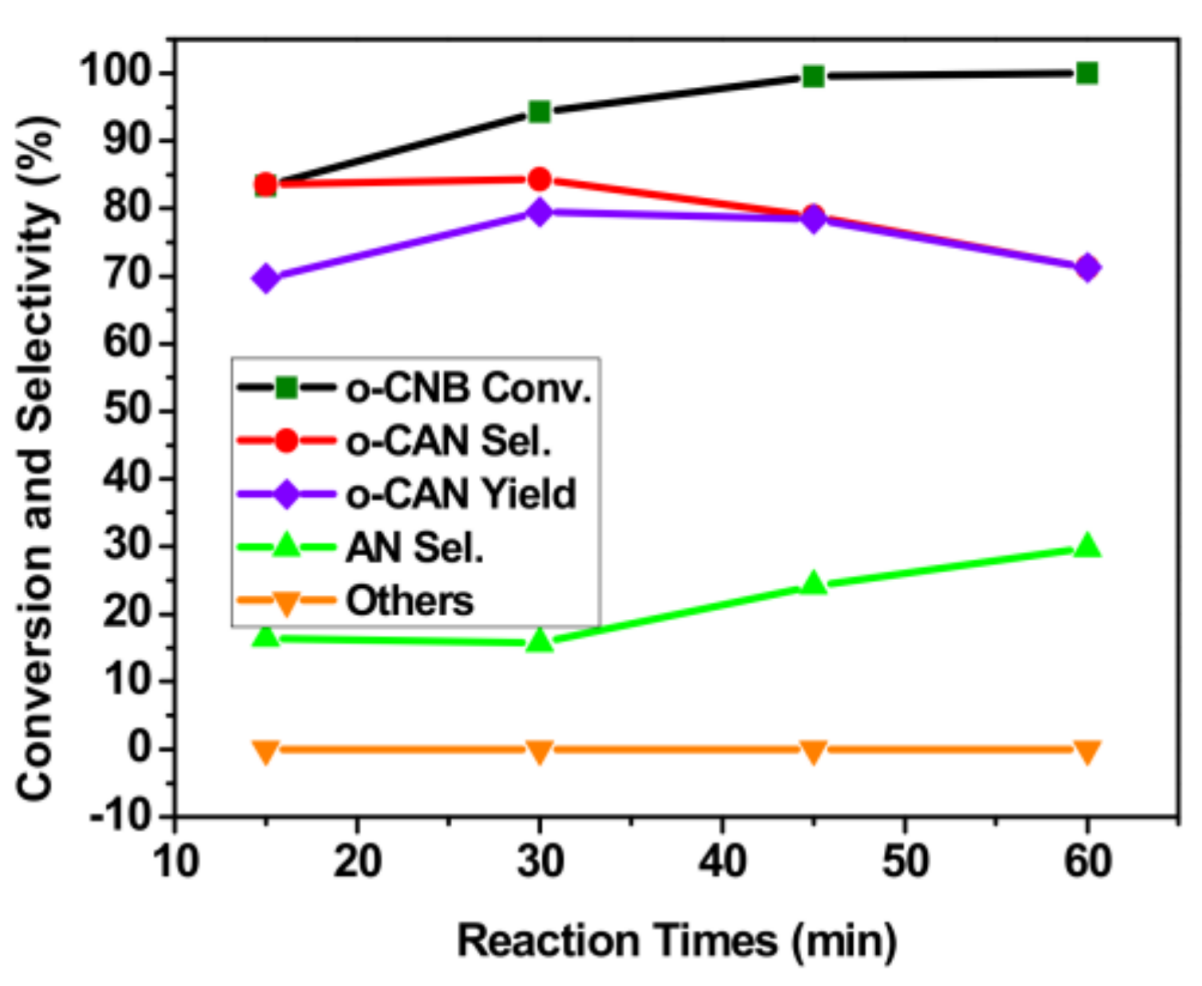
Effects of reaction time on the *o*-CNB hydrogenation over 0.2%Pd/NiO catalyst. Conditions: 60 °C, *P*(H_2_) = 1 MPa, *m*_cat._ = 20 mg, *n_o_*_-CNB_/n_Pd_ = 9000:1, *V*_ethanol_ = 5 mL.

As for almost all of the catalytic reactions, the reaction time course is generally regarded as a vital factor that influences the activity and selectivity [47], and thereby is not neglected. Herein, Figure 8 depicts the variations of *o*-CNB hydrogenation as a function of reaction time. With the increase of reaction time, the *o*-CNB conversion gradually increased to 100%. Two main reasons for the time-dependent increased conversion of *o*-CNB shall be considered here. On the one hand, the interaction between reactants and catalyst becomes more adequate as time constantly lasts, which is beneficial to the hydrogenation reaction. On the other hand, the extension of reaction time further facilitates the transformation of some intermediates (i.e., *o*-chloronitrosobenzene, *N*-(*o*-chlorophenyl)hydroxylamine) to the products mainly *o*-CAN besides dehalogenated product (AN). When compared to those previous studies [48,49], the UV-irradiated Pd/NiO catalyst only required a relatively shorter reaction time (45 min) for the complete conversion of the same substrate (*o*-CNB), which is an energy-friendly case.

The best selectivity of *o*-CAN that was obtained at that time point of 30 min, lower values of *o*-CAN selectivity were given at other time points. Unfortunately, *o*-CAN selectivity almost presents a downward trend in the current range of reaction time. Synchronously, the undesired hydrodehalogenation of *o*-CAN increased slowly with the extension of the reaction time, producing the main by-product (AN). It is probably based on a well-known reason that hydrodehalogenation of halonitrobenzene to nitrobenzene and its hydrogenation to aniline can occur, especially over palladium catalysts [49]. This side reaction shall be the key factor that influences the *o*-CAN selectivity. Besides, the generation of *o*-CAN is possibly inhibited, owing to the further accumulation of more by-products (mainly AN) and intermediates on the active sites when the reaction time is further prolonged. By the way, other by-products except AN is ignored, suggesting that the Pd-based catalyst used here is able to suppress the generation of other by-products to some extent. In other words, this type of Pd-based catalysts over active NiO powder shall serve as potential catalysts for the *o*-CNB hydrogenation if we further improve their ability of inhibiting the hydrodehalogenation of *o*-CAN (note: this is our next work). 

### 3.6. Effect of Reaction Temperature

**Figure 9:**
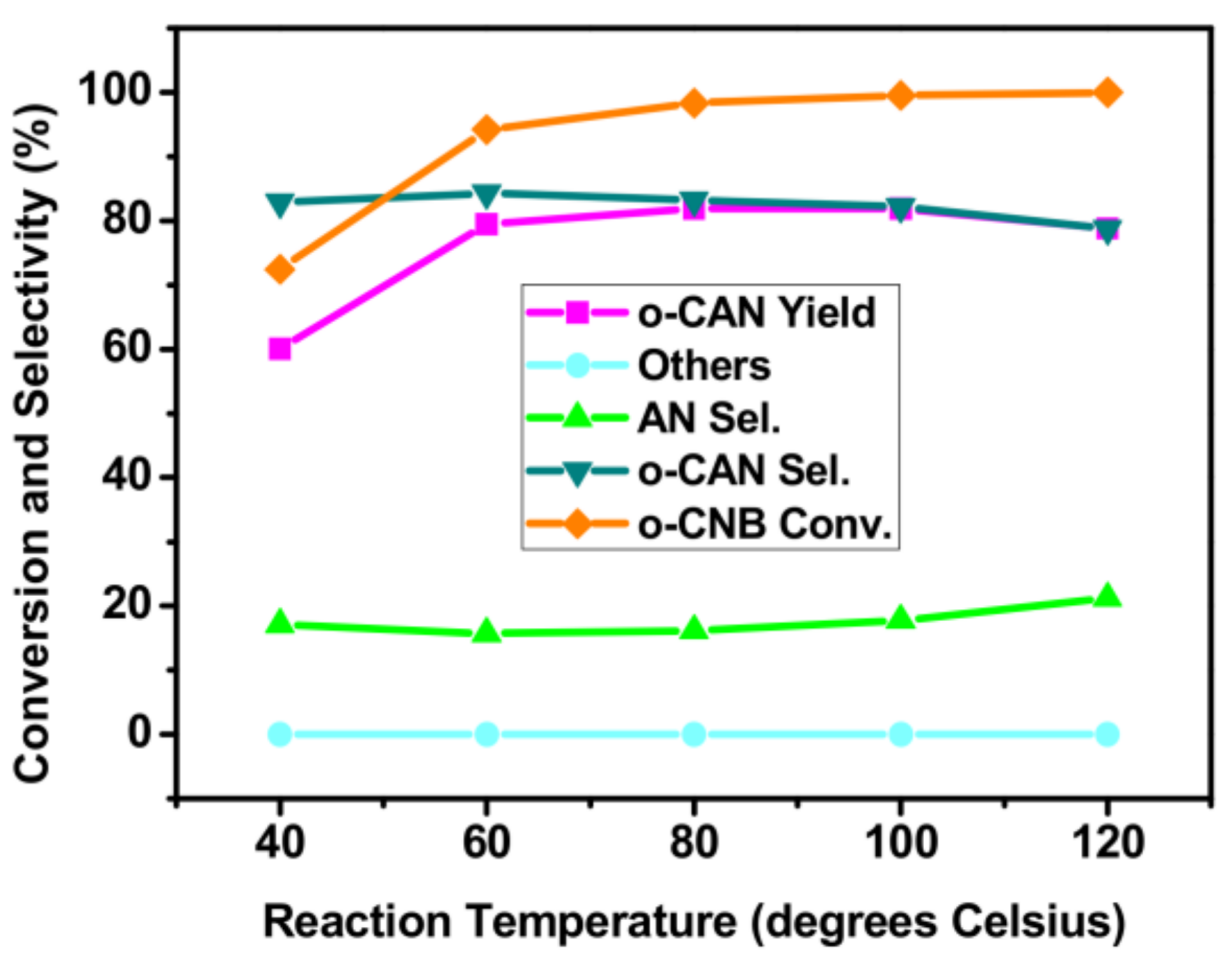
Dependence of the *o*-CNB hydrogenation upon reaction temperature. Conditions: P(H_2_) = 1 MPa, 0.5 h, *m*_cat._ = 20 mg, *n_o_*_-CNB_/n_Pd_ = 9000:1, *V*_ethanol_ = 5 mL.

Figure 9 illustrates the impact of reaction temperature on the catalytic hydrogenation of *o*-CNB over 0.2% Pd/NiO (UV). The *o*-CNB shows sharp increase in conversion in the first operated range of 40 to 60 °C, and then a period of nearly steady-state (approximately 100% conversion) was attained slowly and was maintained at above 80 °C. Moreover, the selectivity of *o*-CAN kept stable value around 83% below 100 °C. Noteworthy, in our case, the high reaction rate of 392.7 L·min^−1^·g_pd_^−1^ was also observed at 60 °C when the hydrogenation of *o*-CNB was catalyzed by Pd/NiO at a high level of *n*_o-CNB_/n_Pd_ ratio (9000). Slight decrease in the selectivity of *o*-CAN was detected as the temperature was further increased. An excess of eighty percent of the *o*-CAN yield reached in the temperature range of 60 to 100 °C. At the same time, elevating the temperature seemed to slightly increase the dechlorination probability of *o*-CNB yielding the main by-product (aniline), meanwhile, the hydrogenation of nitro group occurs. This fact is probably a result of the high intrinsic activity and lower *o*-CAN selectivity [50,51] of the Pd-base catalyst and the temperature-sensitive hydrodehalogenation of *o*-CNB.

### 3.7. Effect of Hydrogen Pressure

**Figure 10:**
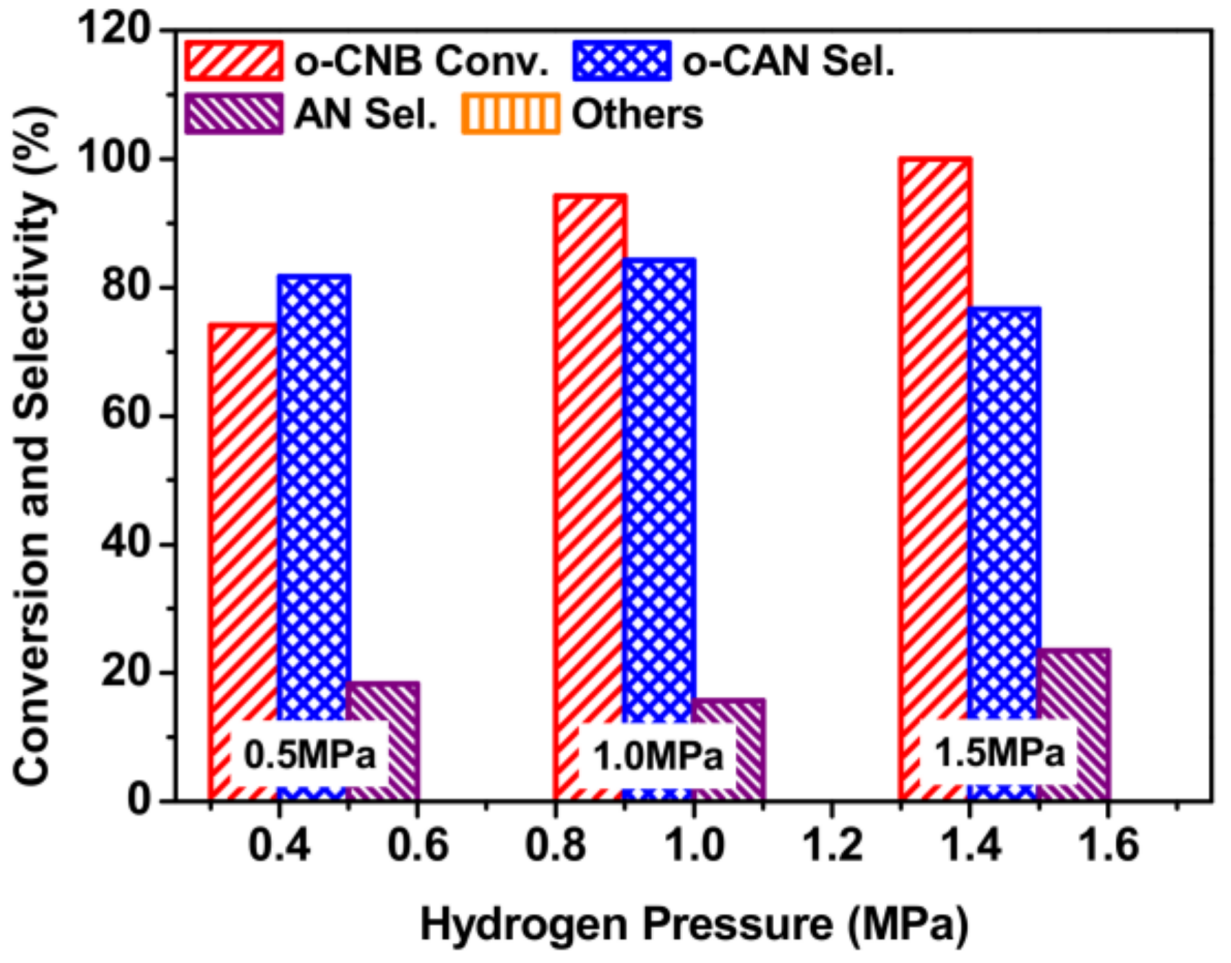
Dependence of the *o*-CNB hydrogenation upon hydrogen pressure. Conditions: 60 °C, 0.5 h, *m*_cat._ = 20 mg, *n_o_*_-CNB_/*n*_Pd_ = 9000:1, *V*_ethanol_ = 5 mL.

The liquid phase hydrogenation reaction is closely correlative to the partial hydrogen pressure [52]. In fact, too high hydrogen pressure does not match with the safety requirements of the actual industrial production, as well leading to more by-products. Hence, in the present work, we only evaluated the hydrogenation behaviour of *o*-CNB under lower hydrogen pressure, i.e., 0.5, 1.0, and 1.5 MPa. The results that are shown in Figure 10 reveal that the *o*-CNB conversion gradually increased with hydrogen pressure, showing a maximum value of 100% at 1.5 MPa hydrogen pressure. This fact is reasonable and comprehensible, which originates from the increasing efficient concentration of soluble hydrogen in ethanol as solvent improving the interaction between the activated hydrogen atom and *o*-CNB molecules absorbed on the catalyst surface. Moreover, the *o*-CAN selectivity located in a region of 81~84 percent under lower H_2_ pressure, followed by a declined tendency with a further increase in *P*_H2_.

Also, experimental observations denote that there is no other by-product (such as nitrobenzene, azobenzene, azoxydichlorobenzenes, and chlorobenzene) except aniline. The above-mentioned fact implies that the 0.2% Pd/NiO(UV) can efficiently compress two main side reactions: (1) the generation of nitrobenzene from the hydrodechlorination of *o*-CNB; (2) the formation of azo compounds that were generated from a condensation reaction of chloronitrosobenzene with chlorophenyl hydroxylamine or o-CAN. As a result, the decreased *o*-CAN selectivity is possibly due to the gradual formation of aniline that results from the further hydrodechlorination [53] of the *o*-CAN and *o*-CNB at high hydrogen pressure. When combining the *o*-CNB conversion with the *o*-CAN selectivity, 1.0 MPa P_H2_ was selected as the operated hydrogen pressure in those evaluations of multi influencing factors on the *o*-CNB hydrogenation over 0.2%Pd/NiO(UV). 

### 3.8. Recycling Test of UV-Irradiated Pd-Base Catalyst

**Figure 11:**
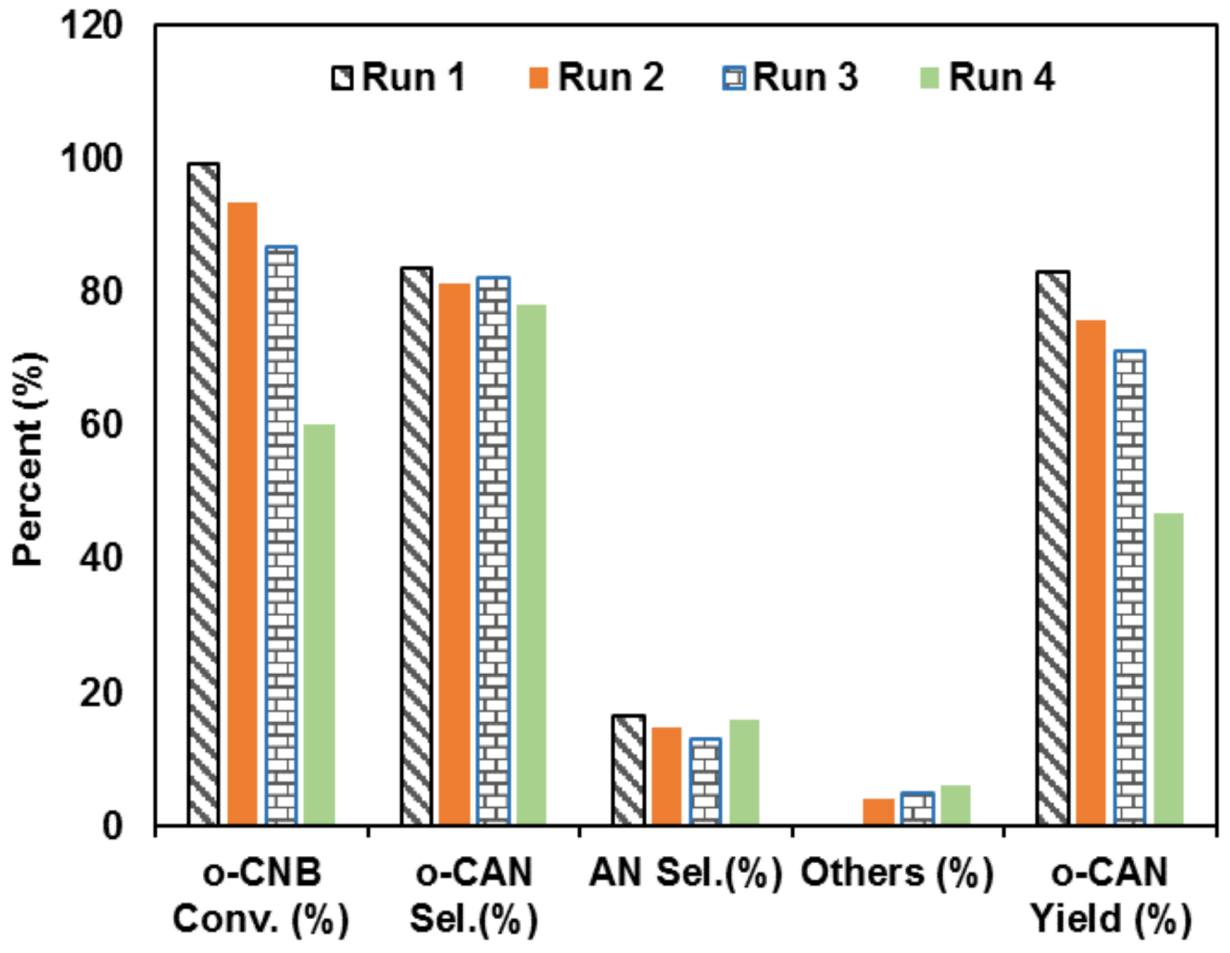
The recycling data of the *o*-CNB hydrogenation over UV-irradiated 0.2%Pd/NiO catalyst. Conditions: *P*(H_2_) = 1 MPa, 60 °C, 0.5 h, *m*_cat._ = 40 mg, *n*_o-CNB_/*n*_Pd_ = 8000:1, *V*_ethanol_ = 5 mL.

The recyclability of UV-irradiated Pd/NiO for the *o*-CNB hydrogenation was investigated with successive runs in the present work. Herein, 40 mg of UV-irradiated Pd/NiO catalyst was used for the *o*-CNB hydrogenation. After each run, the solid catalyst was separated from the reactor by centrifugation and the supernatant liquid was removed. The residual solid was thoroughly washed three times with ethanol, dried in vacuum, and reused for the next run. Figure 11 indicates that the catalyst exhibited better stability and catalytic activity before the fourth run. The *o*-CAN selectivity did not obvious decrease in four cyclic tests. Unluckily, an obvious decrease in the reactivity of UV-irradiated Pd/NiO catalyst was clearly observed in the fourth run, showing excess 36% of decline in activity as compared to the first run. This probably results from four aspects of factors. First, the morphology transformation from mutual ring-linkage structure to separated slices of NiO crystallites, as illustrated in Figure 3D, were likely responsible for the electron transfer between Pd(0) and NiO, leading to the decreasing activity of the UV-irradiated Pd/NiO catalyst. Second, XPS patterns (Support Information Figure S1) of the reused sample represents the oxidation of the in-situ-generated metallic Pd due to oxygen invasion in the operated reaction solution, suggesting the disappearance of active Pd atoms in catalytic system. As compared with XRD patterns that are shown in Figure S3, there is no characteristic peak of metallic Pd for the reused sample. Additionally, the crystallization degree of reused Pd/NiO sample shows a decreased tendency, suggesting the possible aggregation of the catalyst sample in the recycling test, which is in agreement with the observed evidence from the correlative SAED image shown in Figure 5C4. When compared to the initial Pd/NiO (UV) sample, the size distribution (the fourth pattern of Figure S2) of the reused Pd/NiO (UV) catalyst exhibits that the UV-irradiated Pd/NiO catalyst undergoes an obvious aggregation process. Furthermore, the Pd leaching test conducted by ICP confirmed the approximately thirty percent loss of Pd after the third run. Therefore, the as-prepared Pd/NiO catalyst shall undergo a further optimization so that it improves its reusability for practical application.

## 4. Conclusions

The present work reports the performance of Pd-based catalyst over active NiO nanosheets in the hydrogenation reduction of *o*-CNB under varied reaction conditions. UV-light irradiation was proven to successfully induce the dramatical morphology transformation of Pd/NiO sample, which is the key reason for the enhancement (~11-fold difference) in activity of UV-irradiated Pd catalyst relative to the non-UV-irradiated one. Hydrogenation experiments denote that the 0.2% Pd/NiO(UV) catalyst exhibited higher *o*-CNB conversion and *o*-CAN selectivity. Especially, the UV-light-irradiated Pd-based catalyst over NiO carriers still had a good activity for the catalytic hydrogenation of *o*-CNB at a relatively high substrate concentration. Otherwise, it was found that UV-light irradiation or the addition of Pd resulted in interesting change in the morphology of NiO carriers, which probably facilitates the structural modulation of materials. Accordingly, UV-light irradiation is expected to provide an environmental-friendly and economic strategy for the preparation of novel function materials with unique surface morphology. Furthermore, operated reaction conditions for the performance evaluation the as-prepared Pd/NiO catalyst was relatively moderate than most of other previous reports, which is environment-friendly. This property will drive us to carry out fine investigation of influencing factors on the catalytic performance of these catalysts in our future work. 

## Figures and Tables

**Table 1:** Surface parameters of varied catalyst samples with the variations of Pd content.

Catalyst	Surface Area (m^2^/g)	Pore Volume (cc/g)	Pore Diameter (Å)
NiO	25.07	0.178	141.9
0.1% Pd/NiO *^a^*	25.17	0.177	139.3
0.2% Pd/NiO *^a^*	25.29	0.175	138.2
0.3% Pd/NiO *^a^*	25.44	0.173	137.5
0.2% Pd/NiO *^b^*	104.7	0.200	35.9

Notes: *^a^* related Pd/NiO catalyst was prepared under UV-light irradiation; *^b^* the preparation process of this Pd-based catalyst was not under the irradiation of UV-light.

## Supplementary Materials

The following are available online at http://www.mdpi.com/2079-4991/8/4/240/s1, Figure S1: XPS patterns of Pd and Ni elements of as-prepared Pd/NiO catalysts (A) and reused UV-irradiated Pd/NiO catalyst (B). Figure S2: Particle-size distribution of the NiO, non-UV-irradiated Pd/NiO catalyst, UV-irradiated Pd/NiO catalyst and reused UV-irradiated Pd/NiO catalyst. Figure S3: X-ray diffraction patterns of the NiO, UV-irradiated Pd/NiO catalyst and reused UV-irradiated Pd/NiO catalyst.
