# Supplementary material for: UV Light-Assisted Synthesis of Highly Efficient Pd-Based Catalyst over NiO for Hydrogenation of o-Chloronitrobenzene
Source: Nanomaterials (Basel). 2018 Apr 14;8(4):240. doi: 10.3390/nano8040240 (PMC5923570; doi:10.3390/nano8040240)
Supplement: Supplementary file 1 [file nanomaterials-08-00240-s001.pdf]

Supporting Information

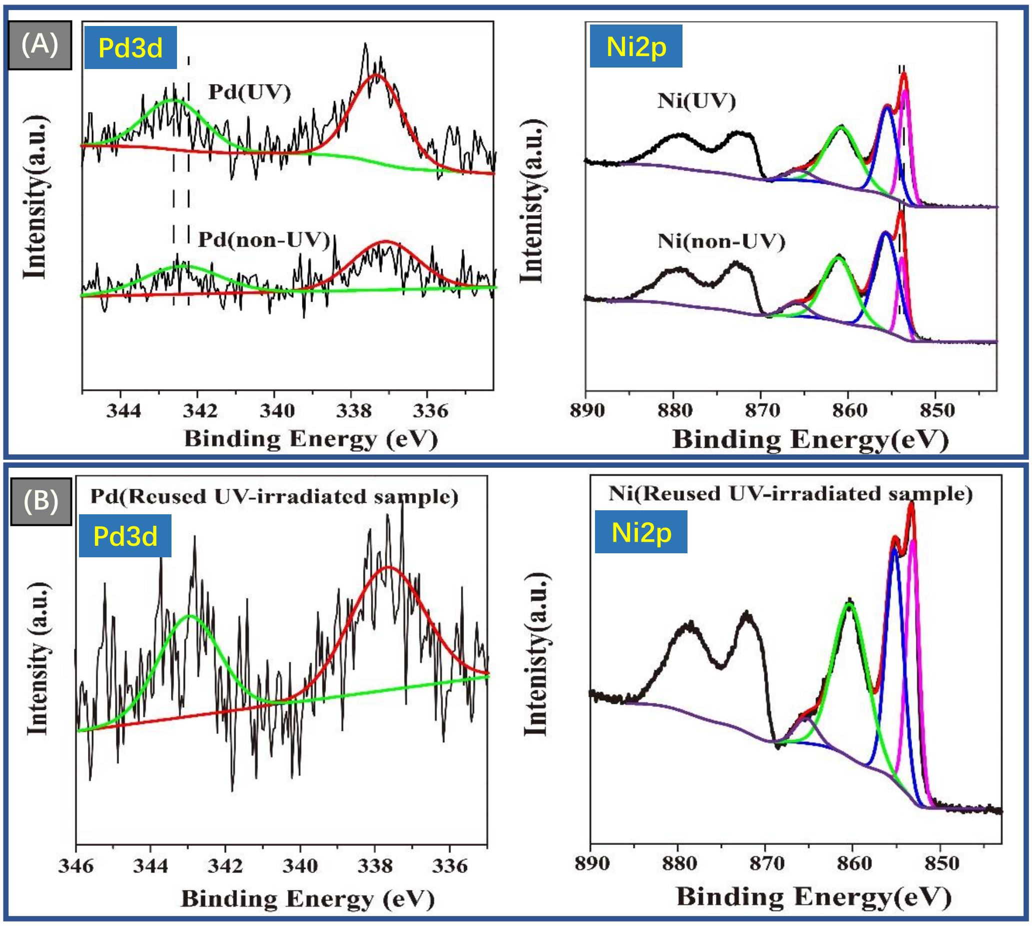

Figure 1S. XPS patterns of Pd and Ni elements of as-prepared Pd/NiO catalysts (A) and reused UV-irradiated Pd/NiO catalyst (B).

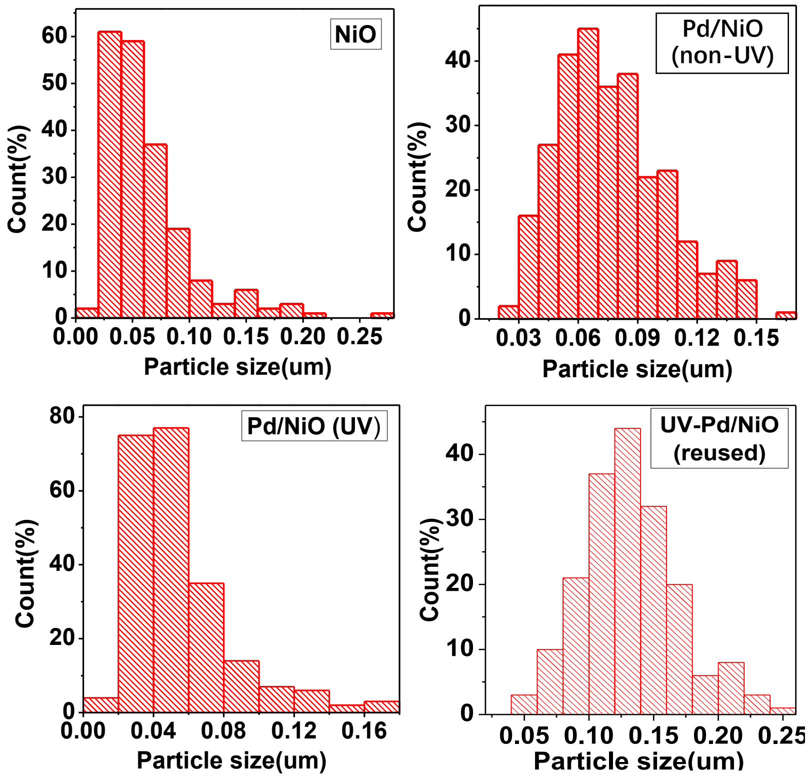

Figure 2S. Particle-size distribution of the NiO, non-UV-irradiated Pd/NiO catalyst, UV-irradiated Pd/NiO catalyst and reused UV-irradiated Pd/NiO catalyst.

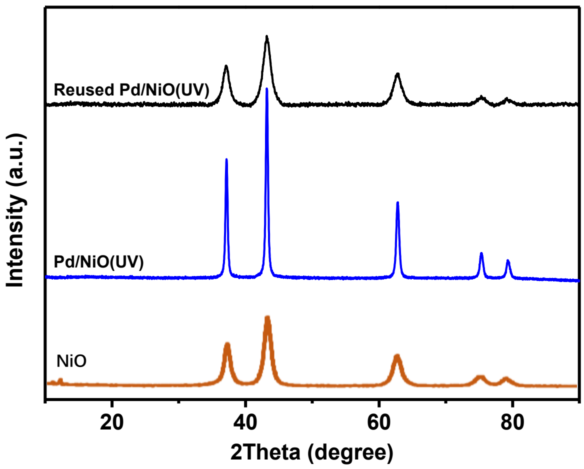

Figure 3S. X-ray diffraction patterns of the NiO, UV-irradiated Pd/NiO catalyst and reused UV-irradiated Pd/NiO catalyst.
